# PRISMA: Program of Research to Integrate the Services for the Maintenance of Autonomy. A system-level integration model in Quebec

**DOI:** 10.5334/ijic.2246

**Published:** 2015-09-23

**Authors:** Margaret MacAdam

**Affiliations:** Factor-Inwentash Faculty of Social Work, University of Toronto

**Keywords:** PRISMA, integration, elderly, coordination

## Abstract

The Program of Research to Integrate the Services for the Maintenance of Autonomy (PRISMA) began in Quebec in 1999. Evaluation results indicated that the PRISMA Project improved the system of care for the frail elderly at no additional cost. In 2001, the Quebec Ministry of Health and Social Services made implementing the six features of the PRISMA approach a province-wide goal in the programme now known as RSIPA (French acronym). Extensive Province-wide progress has been made since then, but ongoing challenges include reducing unmet need for case management and home care services, creating incentives for increased physician participation in care planning and improving the computerized client chart, among others. PRISMA is the only evaluated international model of a coordination approach to integration and one of the few, if not the only, integration model to have been adopted at the system level by policy-makers.

## Background

The PRISMA Research project was established to address the problem of lack of continuity to care experienced by elderly people with chronic conditions in the Estrie region of Quebec. Its objective was to evaluate the implementation of an Integrated Service Delivery Network (ISD French acronym) to improve the health, empowerment and satisfaction of frail older people and to change health and social service utilization without increasing caregiver burden [1]. The features of the Integrated Service Delivery were service coordination, single entry point, case management, individualized service plan, a single functional assessment tool and a shared information system. A unique feature of the PRISMA approach was its coordination and case management feature, which called for the participating agencies to share responsibility for clients but did not require merger of any providers. The PRISMA Project took place in three areas of the region [Sherbrooke (urban), Granit (semi-rural) and Coaticook (rural)].

The PRISMA Project was implemented using a three level governance model with clear responsibility at each level. At the *strategic* level, a local governance table was composed of representatives from the hospitals, the health and social service agency (somewhat similar to provincial home care and case management agencies in other parts of Canada but with a wider mandate), physicians and local community agencies. A major feature of the work of the local governance table was to shift strategic planning from a client focus to a population basis. The local governance table was the “owner” of the project and responsible for its activities. At the *tactical* level, managers of the direct service agencies formed a local management committee responsible for the administration of the project. At this level, staffing issues, budgeting problems and communications among agencies were discussed and adjustments put in place as needed. At the *clinical* level, local clinicians formed multidisciplinary teams to manage client care, ensure the care plan was being followed and to make adjustments to the plan based on client needs.

While all seniors in need were eligible for Project services, the criteria for admission included age (over 65), presence of significant functional disabilities (score over 15 on the functional assessment or be in an Iso-SMAF profile of 4 or over), have support for care at home and need formal care (three or more health care or social services) [2]. There was no financial means test for admission to the Project.

Each geographical area was responsible to manage the total client caseload using its local resources for services, including physician, hospital, residential long-term care and home and community-based care. The local caseload varied by the size of the elderly population in each area.

Services ranged from inpatient, emergency room and outpatient hospital care, primary care, specialized geriatric care, rehabilitation, in-home nursing, therapy, personal care, home support, home-delivered meals, day centres, pharmacy, equipment, supplies and domestic care.

The evaluation of the PRISMA Project followed subjects and their principle caregivers in the three experimental areas in Estrie and in three comparison areas elsewhere in Quebec for four years. Subjects were predominantly women (62%) with an average age of 83. At baseline and throughout the four-year study, the characteristics of both experimental and comparison groups were similar. After four years, the results of the evaluation indicated that, compared to the system of care in the three comparison areas, the PRISMA model produced significant reductions in the prevalence and incidence of functional decline, lowered unmet needs, reduced emergency room visits and had an almost significant effect on reducing hospitalizations. It also found a significant increase in client satisfaction and empowerment. There was no effect on rates of institutionalization, consultations with health professionals or on the use of home care services. There was also no statistically significant change in costs; thus the results of the evaluation indicate that PRISMA was cost effective because it produced improved results at no additional cost [3].

Although the earliest results of the evaluation were not published until 2006, in 2001, the Minister of Health and Social Services required all regions in Quebec to start to implement the PRISMA Integrated Service Delivery features. Each region has flexibility in how they are implemented. Today the PRISMA project does not exist per se; it is part of a Quebec-wide programme called *Réseau de Services Intégrés aux Personnes Âgées* (RSIPA) which has become the normal system of care for the elderly in Quebec.

Early implementation challenges in PRISMA, and now in RSIPA, included gaining tangible cooperation from agency administrators: that is, moving from talking about cooperation to changing agency internal priorities and procedures to make it happen. Operationally, obtaining funding for case management and for a shared information system was critical to implementing PRISMA. These issues were solved for the PRISMA research project but are ongoing problems in the RSIPA programme.

One of the strengths of PRISMA was the sustained commitment of the regional health leadership. Local health and social service leaders formed a partnership among university researchers, the provincial government, regional health and social service planning and funding authorities as well as managers from the home and community care service centres. This group was instrumental in creating the model of care and making resources and staff available to work on it. Perhaps most importantly, key leaders remained in place over a number of years, enabling the integration goal to remain central in the face of financial problems and external changes that could have derailed the project.

Another strength of the PRISMA approach was the adoption of an evidence- and population-based approach. The long-term goal is improved care for all seniors, not just the management of services for the frail elderly.

## PART 1: the model of integrated care

### Introduction

The Program of Research to Integrate the Services for the Maintenance of Autonomy (PRISMA) began with a research project in three parts of the Estrie (known as the Eastern Townships in English) region of Quebec in 1997. The PRISMA Research Project no longer exists because its features have become the regular approach to care of the frail elderly in the Province. The PRISMA approach is now embedded in a province-wide programme called RSIPA or Network of Integrated Services for the Elderly. The province-wide programme is referred to as RSIPA in this report.

### Background

The Estrie region is located 150-km east of Montreal. It has a population of 310,000, with about 13% being aged 65 or over. The region is divided into seven areas; each with its own set of health and social service providers.

PRISMA emerged out of an earlier project in Bois Francs region by a team of researchers at Laval University. This project was designed to alleviate emergency room and other hospital problems in the area. Evaluation results indicated positive results in reducing the desire to institutionalize and in caregiver burden [4]. Features later incorporated in PRISMA originated in this first project.

The objective of the PRISMA Project was to evaluate the implementation of an Integrated Service Delivery Network. The Integrated Service Delivery was designed to improve the health, empowerment and satisfaction of frail older people and to change health and social service utilization without increasing caregiver burden [5].

The PRISMA project was established to address the problem of lack of continuity to care experienced by elderly people with chronic conditions. Lack of continuity affects timely access to care and the efficiency of health care service delivery [6]. Specifically, the local health and social care system exhibited the following problems for elders with ongoing and variable care needs: multiple entry points, services needs determined by the provider rather than by independently assessed client needs, redundant assessments, inappropriate utilization of costly resources, poor hospital to home transitions, delays in receipt of services, poor information sharing amongst professionals and unmet needs [7].

### Client group

The criteria for admission to the PRISMA Research Project were selected based on a review of integration projects for the frail elderly. While all seniors in need were eligible for Project services, only those meeting the criteria below were eligible for intensive case management:

- Age: over 65 years old;
- Significant functional disabilities of a score of over 15 or an Iso-SMAF profile of 4 or higher;
- Support for care at home; and
- Need for formal care: three or more health care or social services [8].

Today, the regions may adjust the eligibility criteria in the RSIPA programme. For example, in the Sherbrooke area, the criteria for admission are stricter: clients must have a risk classification profile of 5 or above, and those under age 65 can be admitted if their abilities and needs meet the criteria for those aged 65 and above (interview).

Clients did not have to meet the Quebec nursing home admission criteria for admission to the PRISMA project and do not have to meet them in the RSIPA programme. There was, and still is, no financial means test for entry to the programme.

There were no formal criteria for discharging clients from the PRISMA Project and there are none today in RSIPA. In Sherbrooke, about 25% of clients are discharged each year because of death or admission to a long-term care facility (interview).

In Sherbrooke, a city in the Estrie region and one of the research areas for PRISMA, there were 3181 clients receiving home care services in 2011. Of these, the average age was 84.6 (ranging from 57 to 106) and 63% were women. Approximately, 40% of home care clients received case management. Using a classification system based on the SMAF assessment data, the RSIPA Sherbrooke client population was categorized as shown in Table 1.

Clients in the PRISMA Research Project, and now RSIPA, are referred to a single entry system but through a variety of mechanisms: they may self-refer, be referred by their physicians or by other professionals in the community, such as hospital discharge planners, home care providers or housing and social care providers.

### Approach to care

The response to the problems (listed above) being experienced by clients, professionals and health system managers in Estrie was to develop an Integrated Service Delivery Network comprised of six main features. These features, evaluated in the PRISMA Project, are now being implemented throughout Quebec in RSIPA.

#### Coordination among services

A distinguishing feature of the PRISMA Research Project and now RSIPA is its emphasis on agency coordination and shared responsibility for clients; it does not require the organizational merger of any providers but it does require agencies to give up some of their autonomy to meet coordination goals.

The foundation for coordination rests upon three levels of governance: strategic, tactical and clinical. See the governance section below for more detail on the stratgetic and tactical levels. At the clinicial level, a multidisciplinary team, composed of representatives of the participating agencies, meets to solve clinical problems. At the time of the PRISMA research project, health and social care providers included hospitals, rehabilitation services, specialized geriatric programmes, public long-term care institutions, private residential care such as retirement homes and group homes, case management, in-home nursing, therapy, social work, equipment and supplies, pharmacy, primary care physicians and other local voluntary services such as home support and day programmes. Today, the local hospitals (with the exception of teaching hospitals), residential and long-term care facilities, and the home and community care agencies (CLSCs) have been merged into one organization, the Centre for Health and Social Services (CSSS French acronym). Representatives of the CSSS meet with the other partners to plan care at the clinical level.

#### Single point of entry

**Figure 1.**
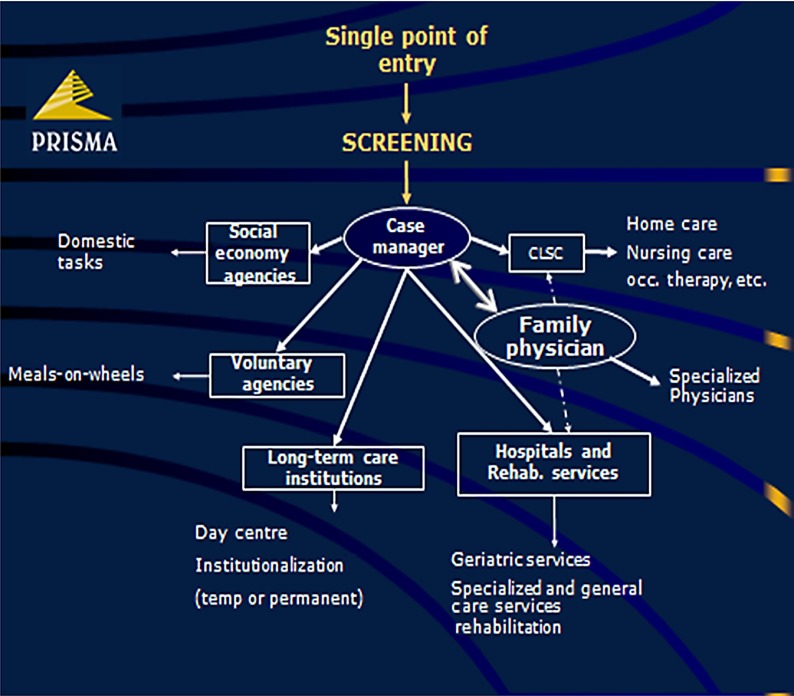
Steps and flow of eligible clients through PRISMA. Source: From Hébert, Raîche, Veil et al. [10].

The flow chart below depicts the steps and flow of eligible clients through PRISMA and now RSIPA (Figure 1). Self-referred clients are screened at the time of initial contact with the programme. A seven question screening tool (PRISMA-7) was developed to quickly identify seniors who should receive the full assessment [9]. A score of 4 positive answers on the PRISMA-7 triggers a referral for a full assessment to determine eligibility for integrated care services.

Those who are not eligble for case management are referred to a local direct service provider. All eligible clients are referred to the case managers.

#### Case management

As is the case with most integrated care programmes, case management was a central feature of the PRISMA programme and now RSIPA. In the PRISMA programme, case managers were staff with special training, were independent of service providers and responsible to the local governance committee. Today, the case managers are co-located with other service providers within the CSSS and may be responsible to the local governance committee or to the CSSS, depending on the region.

The role of the case manager is to perform the basic assessment, develop the care plan based on client needs and family input, coordinate care with the primary care physicians, refer to other professionals as required, ensure that the required services are delivered from participating agencies, advocate for the client as required and conduct periodic re-assessments as part of the monitoring of the client’s situation. The service providers are responsible to the case manager for the delivery of care as described in the care plan although the case manager does not have direct authority over service providers.

The case manager is responsible for managing the full array of services needed by the client and also for maintaining close relationships with the primary care physician who takes on the medical management role for the client. The primary care physician ensures that specialist medical is delivered as needed and coordinates supportive care for medical services, such as transportation with the case manager. All primary care physicians have input to the care plan. Typically, the case manager communicates about the care plan or new medical issues with the primary care physician by telephone or FAX. Currently, most community physicians do not have access to the computerized care plan. If primary care physicians are formally associated with CSSS or the local hospital, they have access to the computerized care plan. Over time, the goal is to enable all primary care physicians in Quebec to more actively participate with case managers.

The case manager is also responsible for managing client transitions from home to hospital and vice versa. If a PRISMA programme patient arrived unexpectedly in the hospital emergency room, the case manager was notified. If the client was admitted, the case manager became part of the discharge planning team to ensure that community supports were available upon discharge. Today, the connection between the case managers and hospital staff can vary from region to region. In Sherbrooke, for example, a list of case-managed clients who have been admitted to hospital within the past 24 hours or visited the Emergency Department is sent to the case management team on a daily basis.

Most case managers are social workers or nurses although other disciplines such as physio- or occupational therapists can take on the case management role. Case managers can provide direct services, such as nursing, but on a very limited basis. The caseload goal is 40–45 clients per worker although it is currently about 50 clients in some areas. There can be wait lists for case management.

#### Individualized service plan

The service plan is developed after the assessment is completed. One of the responsibilities of the case manager is to achieve consensus among other service providers to ensure that the needed services will be delivered in a timely manner. The service providers work with the case manager to help solve issues that arise in adhering to the service plan. Reassessment of the service plan takes place based on the needs of the client; very stable, light care clients may be reassessed less than once a year while clients with very heavy needs are reassessed more frequently. In some CSSS areas, the reassessment process is being revised based on new data on the stability of clients in the classification profiles (interview).

#### Unique assessment tool

The SMAF (French acronym for Functional Autonomy Measurement System [11]) is a 29-item assessment tool used to ascertain functioning in five areas: ADLs, IADLs, mobility, communication and mental abilities. The functional items are based on the WHO classification of disabilities. Data from the SMAF are analysed to produce 14 classification profiles which are used to monitor service planning and usage across similar types of clients [2]. The profiles are also used to adjust admission criteria. In the future, they could also be used to estimate the funding allocation for the programme.

#### Information tool

The computerized client chart is used to track assessment and reassessment data, the care plan and provider interventions. The computerized client chart can be assessed by all professionals and institutions participating in the programme via the Quebec Health and Social Services Network. Strict privacy and security protocols are in place. In addition to monitoring programme operational activities, data from the computerized client chart are available for research studies. An issue with the computerized client chart is that it is not available to physicians in private practice who are not affiliated with a CSSS or otherwise have access to the Province’s Health and Social Service information system. Nor does the current computerized system provide a user-friendly way to input the care plan into the system (interview).

### Service delivery system

Staff at the single entry point emphasize the client’s ability to self-manage their care for those clients not eligible for case management. Clients might be referred to direct service providers such as home care staff within the CSSS or local community partners. When used, partner agencies are reimbursed by the CSSS. The vast majority of clients receive their services directly from the CSSS.^1^

### Quality assurance

As noted above, one of the goals of PRISMA (and now of RSIPA) is improving the empowerment and satisfaction of older people. Case managers place increased emphasis on including client and family preferences in care planning. Today, client/family surveys are conducted every three years to get feedback on satisfaction and experiences with care.

Depending on the area, standardized guidelines for care are used but they are not included in the computerized system.

The *Guide OSIRSIPA* (*Outil de suivi de l’implantation du RSIPA*) measures the degree of implementation of the RSIPA. It is filled out and sent to the Ministry of Health and Social Services once a year and contains a number of process measures such as the extent to which clients receive the services they need, the range of services available and implementation of the PRISMA features.

By 2015, the Ministry expects that an average of 55% of all components of PRISMA/RSIPA will be implemented in Québec (interview). It is expected that the newly appointed Minister of Health and Social Services (the former Dean of the Faculty of Medicine and Health Sciences, University of Sherbrooke and the former PRISMA Research Director at the Centre of Aging) will increase the implementation goal from 55% to 70% (interview).

## PART 2: implementation and organisation

### Implementation

The PRISMA Project took place in three areas of the region (Sherbrooke (urban), Granit (semi-rural) and Coaticook (rural). A comparison group was selected from three areas in other parts of Quebec that had similar characteristics but had not implemented the features of PRISMA. To implement PRISMA, two key features had to be implemented: first; there needed to be adequate funding for case management and second, a shared electronic record, the computerized client chart, needed to be available to the network providers (interview). Both of these challenges were overcome in the PRISMA project but they continue to be issues in implementing RSIPA.

### Governance

A key feature of PRISMA Model is the network model of service delivery. In PRISMA, each provider retains its own organizational and governance independence but the participants recognize the interdependence of the service delivery system and the importance of cooperation in order to improve client and system outcomes. Coordination of care and adherence to the care plan and system goals are supported by three levels of governance.

#### Strategic

During the operations of the PRISMA Project, at the local level, all key decision-makers had a representative on the local governance table. These agencies included the hospitals, the health and social service agency (somewhat similar to provincial home care and case management agencies in other parts of Canada but with a wider mandate), physicians and local voluntary and private service agencies. A major feature of the work of the local governance table was to shift strategic planning from a client focus to a population basis. The local governance table was the “owner” of the project and is responsible for its activities. Prior to the start of operations, the agencies on the local governance table made commitments to support the goals of the programme and to make service and administrative funds available for its operations. If there were serious problems with provider performance that were not solved at either the clinical or tactical levels, it was the responsibility of the local governance table to resolve the issue. Today the local CSSS is responsible for the governance of RSIPA.

#### Tactical

Services managers of the direct service agencies are members of a local management committee which is responsible for the administration of the project. At this level, staffing issues, budgeting problems and communications among agencies are discussed and adjustments put in place as needed.

#### Clinical

Local clinicians form multidisciplinary teams to manage client care, ensure the care plan is being followed and to make adjustments to the plan based on client needs. Physicians are not able to bill for care coordination; they receive no extra financial incentives to participate. Physicians do not normally attend the clinical team meetings but are contacted separately by the case manager if required.

### Organization

Care for the elderly in Quebec is typically delivered through publically funded public agencies, such as hospitals and a variety of not-for-profit and for profit agencies. Case management and service planning takes place within the available health and social care resources of each area. The care package ranges from medical to hospital to rehabilitation to home care services, day programmes and social services such as meals on wheels and home support.

The services provided, the types of agency delivering each service and whether there is a cost to the clients are shown in Table 2.

RSIPA, as was PRISMA before it, is driven by quality and cost concerns. One aspect of improving quality was to include family concerns in care planning and service management. Today, care for the frail elderly is more patient-centred than it has been in the past. Families are actively involved in the care planning process.

### Financial context

The PRISMA project was funded from a variety of sources. Research funding was awarded by the Canadian Health Services Research Foundation, the Canadian Institutes of Health Research, the *Fonds de la recherche en santé du Québec* and *Réseau Québécois de recherche sur le vieillissement*.

During the PRISMA research period, operational funding was available from the *Ministère de la Santé et des Services sociaux du Québec* and the health and social services agencies in the areas of *Estrie, Montérégie, Mauricie et du centre-du-Québec, Laval and Capitale-Nationale CSSS-IUGS (CSSS-Sherbrooke University Geriatrics Institute)* and *Université de Sherbrooke*. Today operational funding for RSIPA is available from the Centres for Health and Social Services (CSSS French acronym).

Clients receive primary and hospital services at no charge under Canada’s universal public health insurance programme covering medically required services. Pharmacy costs are covered under Quebec’s universal pharmacy programme at no cost to clients except for a small co-payment. Professional home care such as nursing is available free to clients while supportive care such as homemaking is provided according to Quebec’s sliding fee programme. Clients receiving residential housing services, such as nursing homes or retirement homes, pay fees depending on their income and type of care they receive. For example, clients living in nursing homes pay for their accommodations while their health, social and medical costs are provided through public funds.

It is important to note that RSIPA operates from the regular allocation of funding from the Ministry of Health and Social Services. When there is insufficient funding for full operations, case management caseloads can become higher than desired and waiting lists for case management and direct services develop.

Accountability for RSIPA spending and results is established through funding and performance contracts between the CSSSs and the regional authorities using measures established by the Ministry of Health and Social Services. If there are shortages of particular services, the regions might decide to make additional funding to expand the service(s). In Estrie, for example, funding of case managers became a priority for the Regional Authority and the CSSSs when new funds were made available by the Ministry.

The Ministry of Health and Social Services has an established rate setting mechanism for provider remuneration, which establishes payment rates for RSIPA services.

## PART 3: impact and sustainability

### Evidence of impact

The evaluation of the PRISMA Project followed subjects and their principle caregivers in the three experimental areas in Estrie (Sherbrooke, Granit and Coaticook) and in three comparison areas from the Chaudière-Appalaches region for four years. Subjects were predominantly women (62%) with an average age of 83. At baseline and throughout the four year study, the characteristics of both experimental and comparison groups were similar. After four years, the results of the evaluation indicated that, compared to the normal system of care, the PRISMA model produced significant reduction in the prevalence and incidence of functional decline, on meeting unmet needs, and reduced Emergency Department visits and had an almost significant effect on reducing hospitalizations. It also found a significant increase in client satisfaction and empowerment. There was no effect on rates of institutionalization, consultations with health professionals or on the use of home care services. There was also no statistically significant change in costs; thus the results of the evaluation indicate that the Project was cost effective because it produced improved results at no additional cost [3].

At the point at which the PRISMA Project reached 85% of clients and service providers, it was estimated that implementation costs were about $27 per person aged 65 or older in urban areas and from $37 to $47 per senior in rural areas (2002 dollars). Operational costs per case ranged from $1811 in urban areas to from $1570 to $2246 in rural areas at an average caseload management of 35 cases per worker. The expectation was the costs would fall as the case managers took on full caseloads of 45 cases [12].

Mean annual costs in 2002 dollars, including continuing care services (home care and institutional care, hospital care, daycare, respite, medical care, medication costs and the integrated care network) costs for the experimental group, varied from $17,870 to $20,466 between the first and fourth year in the experimental group and $17,963 in the first year to $20,734 in the fourth year in the comparison group (all public, private and volunteering costs included). Statistical analyses showed no significant difference between the two groups for total annual costs. Researchers concluded that the PRISMA model is more efficient considering the outcomes on functional decline, satisfaction, empowerment and unmet needs; as efficient considering functional autonomy; and less efficient considering social functioning and caregiver burden [13].

The positive results of PRISMA were not reached until year 3 of the evaluation. By year 3, the rate of implementation of all of the key features of the model in the research areas approached 80%. The rate of physician participation was 73% [12].

Today, it is not possible to compare outcome and cost data because the features of the PRISMA project are being implemented in the RSIPA programme across the province. Results from the PRISMA project indicated that effects of the network are felt when the implementation level reaches 70%. Thus the Ministry of Health and Social Services is placing emphasis on the regions to reach at least 55% implementation by 2015 (interview). Some consider this goal as too low and expect it to be raised (interview).

### Sustainability and spread

As mentioned above, PRISMA features are now embedded in RSIPA, which is becoming the normal system of care for the frail elderly in the Province. The decision to adopt the PRISMA model across the province is the key test of sustainability of any demonstration.

The province is currently monitoring the degree of implementation of the RSIPA features in every region. From 2008 to 2011, there has been impressive improvement in implementing the key features of the programme as can be seen from Table 3.

It can be seen that there has been little progress in implementing the service plan. Every client has a service plan but the issue reflected in this chart is that the service plan cannot be added to the shared computerized record in a user-friendly manner.

Performance measures are shared among the network providers. There are no financial or other special incentives to reach outcome targets. If there are shortages of particular services, the local governance board might decide to make additional funding to expand the service(s). In Estrie, the board has made additional funding for case management and home support services, for example (interview).

Additionally, there is a PRISMA-type replication being implemented for frail elderly people underway in France; thus the model may spread in some form to at least one other country [14].

## PART 4: barriers and facilitators to effective implementation

### Systemic and contextual factors

#### Leadership

A key feature of the PRISMA Research Project was the partnership among university researchers, the provincial government and regional health and social service planning and funding authorities as well as managers from the home and community care service centres (CLSCs French acronym). Two research teams (Research Centre on Aging in Sherbrooke and Laval University Geriatric Research Team in Quebec City), the Ministry of Health and Social Services, the Health and Social Services Boards in Estrie, Mauricie et du Centre-du-Québec, Laval, Montérégie, Capitale-Nationale and the Sherbrooke Geriatric University Institute came together to plan, implement and evaluate the effectiveness of the initiative. The importance of this partnership cannot be overestimated because, in addition to supporting the design, implementation, operations and evaluation of the PRISMA project, it was instrumental in simultaneously moving an integrated care agenda ahead throughout the entire region. *The work was promoted and sustained for over 15 years through the commitment of the key leaders in the Region*.

#### Moving beyond talking to action

From the outset, the PRISMA project, operating in three areas of the Estrie, was embedded within a health system culture that was working to improve integrated care for the elderly population throughout the region. However, it took time to move from talking about cooperation for agencies to change their procedures. Without the sustained leadership and focus of key players, the development of shared accountability for care would not have happened (interview).

PRISMA/RSIPA is a coordination model which depends on the ability of local providers to voluntarily participate in the programme by giving up some of their own autonomy. The PRISMA Research Project built upon efforts to increase coordination of services in the Estrie Region which began in 1995, when, in six of the seven areas of the region, the local hospital, the home and community care service centre and residential and long-term care centres merged to form a single entity. In 1998, the Estrie Regional Health Authority recommended implementing integrated networks across the region (which were being tested in the three PRISMA research sites in Estrie). These networks were to include a joint triage system, a case management approach and the use of individualized service plans. Over time, the key elements of integrated networks were put into place as recommended by the researchers involved with the design of the PRISMA project. During this period, the Quebec Ministry of Health and Social Services implemented changes designed to encourage integrated care for the elderly in the Province. For example, the Ministry issues guidelines for coordinated care of the elderly in 2001; in 1998, the home and community care centres (the CLSCs) and later other agencies started to use a Multiclientele Assessment Tool (OEMC French acronym); and centralized single entry points for care in each of the areas were established in 2001. The use of a common assessment instrument allowed the transmission of client data among providers. In 2001–2002, case management was implemented in the region. In 2002, the PRISMA-7 instrument was put in place to locate frail elders [15].

In Estrie, PRISMA was not viewed as a one-time research project designed *only* to test research hypotheses but rather as part of a larger system change designed to improve care for the frail elderly.

#### Flexibility at the regional and local levels

One of the strengths of the implementation of the changes in Estrie, including the PRISMA project, and now RSIPA has been the understanding that a range of local and regional differences affect implementation of policy goals. For example, it took longer to implement the PRISMA features in the rural demonstration area than in the urban and semi-rural areas. A key decision has been to allow regions flexibility in implementing the features of RSIPA in ways that are appropriate for local areas.

### Organizational factors

The health and social care governance structure in Quebec is already more integrated than in other Canadian provinces. Quebec is the only province in which all social and health care services are administered by one Ministry.

#### Reorganization

There was an externally mandated reorganization of the entire health care system in Quebec in 2004. The reorganization affected the pace of implementing integrated care in Estrie and the model itself. It is still impacting the system in Quebec.

In 2004, the Quebec Legislature passed Bill 25, an Act Respecting Local Health and Social Services Network Development Agencies which called for the reorganization of the health and social service system in the Province. The reorganization called for three levels of governance:

*Regional*. Eighteen regional agencies are responsible for planning, coordination and allocation funding for the local system of care;

*Area networks*. Within the regions, local health and social service networks were created which include all health and social care providers at the local level and are accountable for the health and social care of the population within their area; and

*Health and social care centres*. Each area network created a health and social care centre from the merger of the local hospital(s) (teaching hospitals were exempted), publicly owned long-term care facilities and the home care programme (CLSC). The Centres (CSSS) are the focal points of care within each network but they work in partnership with other agencies in the network. There are now 95 health and social service networks and centres (CSSS) across the Province.

The extent of the merger temporarily diverted the energy of health care managers away from ongoing integration or other activities to deal with the governance, human resources, clinical care and funding repercussions of the merger. This was just as true in Estrie as it was elsewhere. The merger activity is proceeding at different speeds across the Province; today some areas have completed the merger while others are still in process.

The results of the merger have been both positive and negative in Estrie. On the positive side, there are fewer different agencies involved in service coordination and decision making at the strategic, clinical and tactical levels: thus problems can be resolved more quickly. But, there is concern about the balance of power between organizations of different sizes and budgets. An ongoing issue is that agencies outside of the CSSS may not receive adequate funding to support their services (interview). As well, the merger upset the established relationships amongst providers; new relationships are being developed (interview).

Even with the merger, there is still silo budgeting for individual types of services (interview). The merger might make it easier to implement new funding models, such as capitation, that could encourage resources to be used more efficiently.

During the period of the merger, clinical integration was set back because of the time and resources needed to create a new culture and operations within the newly merged organization. Researchers associated with PRISMA stress that mergers are not necessary for the model to be successful (interview).

Some of the other possible advantages of the merger have yet to be realized In addition to the funding possibilities. The merger might help to reorient the system towards a more preventative approach. One researcher suggested making *aging per se* the focal point for planning and funding at the strategic level (interview).

### Operational/service delivery factors

- *The model features*. The PRISMA model places emphasis on service coordination, single entry point, case management, individualized service plan, the unique assessment tool and the electronic information tool. Researchers and managers feel that each feature is essential to the overall success of integrated care for the frail elderly (interview). As the model has matured, it has become more evident that service partners need to include areas outside of the Ministry of Health and Social Services, such as the Ministry of Transportation and the Ministry of Municipal Affairs (interview).
- *Information systems*. Another key externally mandated change which affects the delivery of care has been the implementation of a new province-wide information system, *SOLUTION RSIPA*. Its’ platform is more stable than the earlier system used for PRISMA but it does not yet carry all of the features available under the earlier system. For example, it is not possible to enter the Service Plan into the system in a user-friendly manner. All key players stress the importance of a shared electronic record to promote integration and the need to allocate funding to improve the *SOLUTION RSIPA* (interview). The Ministry is expected to have improved the shared electronic record, especially with regard to the ease of entry of the care plan and medical evaluation by April 2014 (interview).
- *Unmet needs*. The province has not made new money specifically available for case management (interview). As a Province, over time, Quebec has made proportionately less funding available for home and community care than other provinces [16]. As a result, there can be waiting lists for case management and home care services. The size and type of waiting list varies across the Province depending on local conditions and decisions made within the region and in the local CSSS. For example, in Sherbrooke, there is a waiting list for case management and home care services. Work is underway to optimize case management and home care services by regrouping and reorganizing them according to the level of service intensity required [low intensity, short term, high intensity (case management)]. To manage high need care, the single point of entry team ensures that services that are immediately required are put in place while the client is on the waiting list for case management. If needed, the single entry point staff will also do follow-ups (“*relances*”) during the waiting period (interview). Other regions may be taking similar steps to manage the demand for care with available resources. The Ministry is working to assist regions to reduce wait times for services and to help them shift more care in the community and away from hospitals and long-term care institutions (interview).
- *Physician remuneration*. Most primary care physicians in Quebec are paid on a fee-for-service basis which does not reimburse them for their time in care planning and service coordination. Even though in the PRISMA Project, 73% of physicians participated in the new model of care for their frail patients [17]; a new reimbursement system could increase their level of activity (interview). The Ministry is expecting to have implemented new agreements with physicians regarding both their ability to make home visits and to interact with case managers by the summer of 2013 (interview).
- *Training and coaching*. The pace of change and staff turnover can quickly result in redirection of staff at all levels away from the goals of RSIPA or any other system change. Senior managers stress the importance of ongoing coaching and training about the goals of the RSIPA model. It is not enough to train staff at the onset of a change; managers need to be aware of the need to plan for the time and resources to maintain staff adherence to the goals of the change (interview). At the case management level, it is not an automatic activity of health professionals to consider the possible role of other providers, especially those at the home support level. There is a need to train case managers in the coordination features of their roles in RSIPA’s integrated approach to care. Ongoing coaching is required to ensure that care plans reflect the full range of appropriate services, especially those that can be provided by the smaller community agencies (interview). The Ministry is seeking ways to assist the regions with staff training and coaching such as by producing training tools that can be used across the province. Some of these tools might also address best practice issues and coordination of services (interview).
- *Case management*. In Quebec, case management for the elderly had been seen as a social work role. In PRISMA, other disciplines, such as nurses and rehabilitation therapists became case managers which provided an interdisciplinary approach. In other regions, there has been resistance by social workers to including other disciplines as case managers (interview). At the moment there is no one model of case management in Quebec. Researchers are working with the Ministry to develop a uniform case management model which may be completed in June 2013 (interview).
- *Quality management*. The approach to quality assurance in Quebec has been to focus on process measures because it is very difficult to attribute outcomes to any particular intervention when so many variables are involved. At some point, it may be possible to compare the impact of RSIPA on other systems of care for the elderly in other jurisdictions.

## Conclusions

The results of the PRISMA Project directly influenced public policy relating to the care of frail elderly people in Quebec. The key factors for success were rooted in the composition and role of the group at the strategic level which not only provided sustained leadership but also included researchers who grounded the features of the intervention in a thorough review of the integration literature. Their recommendations were supported by local decision-makers who were motivated to improve the quality of care as well as to contain costs (interview).

The basic features of the PRISMA model are not very different from those used in many other integration efforts. But PRISMA did not require either vertical or horizontal mergers or the creation of a new or greatly modified entity, such as the PACE projects require. It remains the only example of an evaluated coordination-type model as described by Leutz [18].

One of the lessons learned from other integration projects is that they need time to develop because of the range of service providers (from the very large, such as hospitals to the very small, such as local home delivered meals programmes). The PRISMA evaluation was a four-year longitudinal study. The wisdom of the choice of four years was seen in the fact that it took until year 3 to reach a 70% implementation rate of all the features. The impact of the model was not apparent until year 3.

There are 10 provinces in Canada and three territories (the material below does not include information about the territories). All provinces are implementing PRISMA features to varying degrees although not necessarily because they were influenced by PRISMA. Almost every province has a single entry point and case management service that includes assessment, care planning, ongoing monitoring and re-evaluation. Eight also have coordinated discharge planning with hospitals. However, most do not have shared electronic information systems. Nor do they have strong linkages with primary care physicians [19]. Administratively, as noted above, only Quebec administers all health and social services under one Ministry. The differences between the approach taken in Quebec and the rest of the provinces are:

- Implementing mechanisms for strong coordination across the range of service providers, include primary care physicians and hospitals;
- The emphasis on a population-based approach, not just the management of services for the frail elderly.

These last two features are unique strengths of PRISMA and now RSIPA.

A feature of many case management models such as PRISMA/RSIPA is the separation between efforts to plan and deliver a wide range of health and social services to clients with chronic conditions and efforts to improve medical care for these same individuals. In Canada, reform of primary and specialized medical care has been viewed by policy-makers as independent of the local community care system. Because of issues in Canada related to waiting periods for care, primary care reforms have largely focused on increasing the supply of primary care physicians and on efforts to more efficiently manage patient loads, although there are chronic disease improvement projects such as those for people with diabetes, heart disease or Alzheimer Disease, to name a few. These projects tend to be led by specialist physicians supported by other health professionals but usually have weak links to case managed community care programmes. Yet both the community care programmes and the disease-based medical programmes typically have the same goals: to improve access to and quality of care for patients/clients and to be cost effective, usually by providing more care in the community. It would appear that a next step for RSIPA in Quebec and for other somewhat similar efforts in other parts of Canada would be to strengthen linkages between primary care and chronic disease reform with case managed community care reform to create truly integrated chronic care programmes.

## Figures and Tables

**Table 1.**
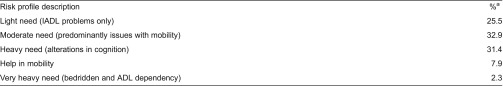
Distribution of population risk classification profiles: Sherbrooke 2011

^a^The percentage of those who were assessed in 2011 in Sherbrooke

**Table 2.**
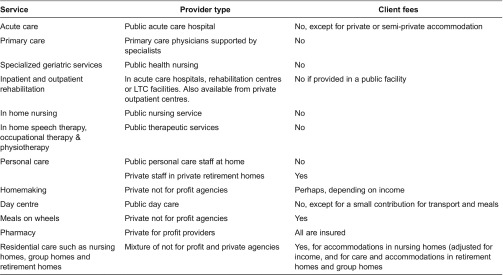
Services, provider type and client costs

**Table 3.**
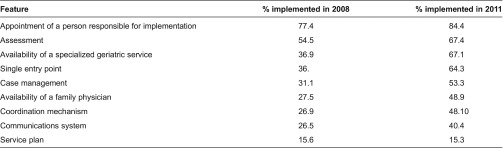
Degree of implementation of network integration features in Quebec: 2008–2011

Source: CSSS Estrie (2012)

## Note

^1^​ In some regions, a direct payment option is available for certain clients. In Sherbrooke, clients with decreasing independence, low income and living in private retirement homes can receive a direct payment option which allows them to organize their own care providers. Otherwise, they receive services under the usual system (from the CSSS and/or community partners). A full assessment is required. If clients opt for the direct payment option, usually they use the payment to pay for extra care from the staff of the retirement home. Clients receiving the direct payment option are followed by a case manager or other staff (if they do not meet case management criteria). They are assessed annually or more frequently as required. In all cases, the wishes of the client and family are part of the care planning process (interview).
